# Multi-contrast magnetic particle imaging for tomographic pH monitoring using stimuli-responsive hydrogels

**DOI:** 10.1038/s44172-026-00586-8

**Published:** 2026-01-17

**Authors:** Bruno Kluwe, Justin Ackers, Matthias Graeser, Anna C. Bakenecker

**Affiliations:** 1 Fraunhofer IMTE, Fraunhofer Research Institution for Individualized and Cell-Based Medical Engineering, Lübeck, Germany; 2 Physikalisch-Technische Bundesanstalt, Berlin, Germany; 3 Chair for Metrology, University of Rostock, Rostock, Germany; 4 Medical Engineering, Department of Electrical Engineering and Information Technology, Technical University of Darmstadt, Darmstadt, Germany

**Keywords:** Biomedical engineering, Imaging techniques, Biomaterials, Sensors and probes

## Abstract

Magnetic particle imaging (MPI) is a tomographic imaging technique which determines the spatial distribution of magnetic nanoparticles (MNPs). Multi-contrast MPI provides the ability to detect environmental conditions of MNPs, such as temperature or viscosity. One parameter that has not been investigated but shows high potential for medical diagnosis is the pH value, as it is an indicator of inflamed or tumorous tissue. In this work, we present an approach to resolve the pH value using multi-contrast MPI. Our proof-of-concept is based on a stimuli-responsive, magnetic hydrogel that exhibits reversible swelling in response to a pH change. The pH contrast is generated indirectly via the pH-responsive hydrogel swelling modulating the signal of embedded MNPs. Magnetic particle spectrometry measurements show that the hydrogels’ magnetic response correlates with the pH value, which could provide a new way of contactless pH monitoring. Finally, the feasibility of resolving different pH values in a multi-contrast MPI image is demonstrated.

## Abstract (web summary)

Bruno Kluwe and colleagues propose using stimuli-responsive, magnetic hydrogels in magnetic particle imaging to resolve pH levels. Non-invasive pH measurement is promising for diagnosing inflamed or tumorous tissues.

## Introduction

Magnetic particle imaging (MPI) was first described in 2005 as a three-dimensional tomographic imaging technique that enables non-invasive measurement of the spatial distribution of magnetic nanoparticles (MNPs)^1^. In contrast to magnetic resonance imaging, MNPs do not act as a supporting contrast agent, but are the only source of magnetic signal in MPI, which enables direct quantification^2^. As a related technique, magnetic particle spectrometry (MPS) was developed as a zero-dimensional spectroscopic version of MPI^3,4^. Using MPS, it was possible to determine the temperature of magnetic particles^5^. Following this milestone, multi-contrast MPI emerged as a new research field that expands the capabilities of MPI, offering new functionalities in addition to imaging the spatial distribution of MNPs^6^. A multi-contrast reconstruction approach provides sensitive information about the environmental conditions of MNPs, enabling not only spatial temperature profiles^7^ but also viscosity mappings^8,9^ of a magnetic nanoparticle (MNP) solution. Other groups also showed that MPI enables the distinction of different MNPs by discriminating different core sizes^10^. Estimating the spatial orientation of immobilized MNPs with parallel-aligned easy axes is also possible by simultaneously performing spatial detection of MNPs^11^. In addition, using MPI, it is possible to discriminate between free and cell-bound MNPs, demonstrating the high potential of MPI as a cell imaging technique^12^. Using these different approaches, MPI proves to be a powerful tool for investigating magnetic nanoparticles and their environment^13,14^. One potential medical application of multi-contrast MPI includes functional neuroimaging, thereby enabling visualization and differentiation between areas of liquid and coagulated blood^15^. Together with a human head-sized MPI scanner, multi-contrast MPI shows great potential for clinical application in the diagnosis of ischemic strokes and intracranial hemorrhage^16^. Another medical application lies in the use of multi-contrast MPI for cardiovascular interventions^17^, including, for example, image-guided catheter steering^18^ and stent positioning^19^.

The objective of this research is to resolve pH in multi-contrast MPI, which has not been investigated before. The pH value is known to be an important parameter for medical diagnosis, as certain human diseases, such as infections, injuries, inflammations, and solid tumors, are associated with altered pH values^20^. Determining local pH values can provide additional biochemical information that can support the diagnosis and monitoring of these conditions related to a pH change^20,21^. Consequently, resolving the pH values of different environments would be beneficial when it comes to the clinical application of MPI. In this research, the pH value is resolved in MPI by performing pH mapping through a multi-contrast reconstruction. The foundation of the presented approach is a chemically stimuli-responsive hydrogel that undergoes a volume change in response to a local pH alteration. First presented in 1960, hydrogels are versatile polymer networks that have attracted increasing scientific attention since the late 1990s^22,23^. Due to their mechanical properties and biocompatibility, hydrogels are used for a variety of biomedical and biotechnological applications^24–26^. However, one of their most advantageous characteristics is their ability to respond to external stimuli, making them a promising material for a wide range of sensing applications^27–29^. Arifuzzaman et al. developed an implanted pH sensor based on 2D plain radiography to detect implant-associated infections. The underlying principle of the sensor was also based on the pH-dependent swelling of a hydrogel, where volume alteration was measured radiographically by detecting an indicator pin on an integrated scale^30^.

We realized the read-out of the pH value without the need for ionizing radiation. In this proof-of-concept study, the pH contrast is generated indirectly through a change in the measured MPI signal of MNPs embedded in a pH-responsive hydrogel patch. Before MPI is performed, the swelling behavior of the patches is investigated by measuring the swelling ratios at different pH values. Then, the signal behavior of the magnetic hydrogel patches has to be determined. This is accomplished through examination of the MPS amplitude spectra of the patches in dry state and in various swollen states, which is achieved through immersion in solutions with differing pH values. Furthermore, vibrating sample magnetometer (VSM) measurements are conducted to examine the magnetization characteristics of the patches. The final objective is to demonstrate the feasibility of resolving different pH values in MPI using a multi-contrast reconstruction approach. Therefore, an MPI-scan of a phantom containing hydrogel patches in different pH solutions was performed. Using a multi-contrast reconstruction, the different pH values can be differentiated through a color mapping in the reconstructed image.

## Results and discussion

### Principle of resolving pH

The pH value is coupled to the MPI signal by using a stimuli-responsive hydrogel with embedded MNPs. In response to alterations in the pH value of the surrounding solution, the pH-responsive hydrogel undergoes a change in volume that is manifested as the absorption and release of the surrounding solution. This pH-dependent volume change is caused by anionic groups, functional groups of acrylic acid integrated into the hydrogel matrix, which ionize depending on the pH value. This behavior generates repulsive forces that cause swelling of the gel and an increase in volume in higher pH values and a shrinking of the gel at lower pH values^31^

**Fig. 1:**
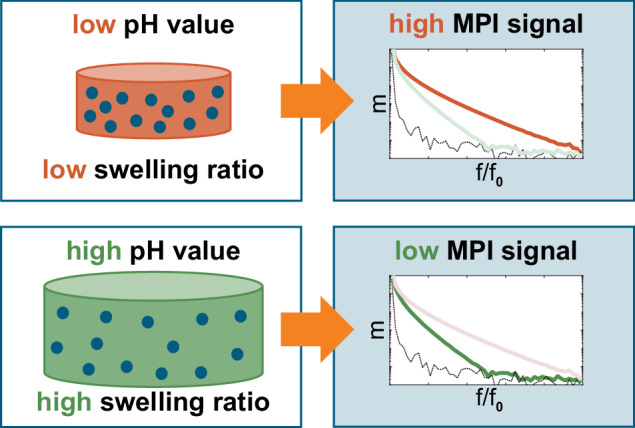
Principle of signal generation for resolving pH in magnetic particle imaging using a pH-responsive magnetic hydrogel. A pH change causes a reversible hydrogel volume alteration, which results in a modified MPI signal. In this way, a certain magnetic signal can be assigned to a swelling ratio and thus to a certain pH value.

In order to facilitate the detection of this volume change magnetically, superparamagnetic iron oxide nanoparticles (SPION) are embedded in the hydrogel matrix, thereby integrating magnetic properties into the hydrogel. Inside a solution with a low pH value, the hydrogel exhibits minimal swelling, resulting in a high signal intensity (see Fig. 1). In contrast, at higher pH values, the hydrogel undergoes volume expansion, leading to a reduction in the magnetic signal. The use of this effect facilitates the detection of swelling through a change in the magnetic signal, which can be quantified using MPI. The resulting signal can then be correlated with a pH value.

### pH-responsive magnetic hydrogels

**Fig. 2:**
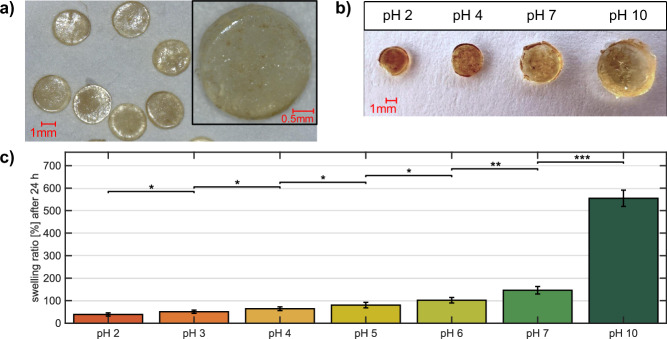
Magnetic hydrogel patches and their pH-dependent swelling behavior. **a** Uniformly cured hydrogel patches with embedded Synomag magnetic nanoparticles in the dry state. **b** Magnetic hydrogel patches in different swollen states in pH 2, 4, 7, and 10. **c** Mean swelling ratio for magnetic hydrogel patches after 24 h in different pH solutions compared to the dry state. Error bars denote standard deviations with *n* = 6, statistical significance indicated with stars.

Figure 2a shows the magnetic hydrogels in a dry state using reflected light microscopy. The photocured cylindrical patches are fabricated from a synthetic copolymer hydrogel, and SPIONs are integrated through particle absorption in SPION solution of Synomag-D particles. The UV-photopolymerization process is a reliable and straightforward method for curing hydrogels in the millimeter range. The process enables the synthesis of a substantial number of hydrogel patches in a short time frame under mild reaction conditions (without heating or extreme pH conditions). Furthermore, immersion of hydrogels in MNP solutions is a convenient method to incorporate particles into the gel. In addition to incorporating MNPs through swelling into the hydrogel, MNPs are also mixed with the hydrogel solution before curing. For this manufacturing process, it was found that the magnetic signal of the patches reduces, probably because of stronger binding of the particles into the hydrogel matrix, leading to increased immobilization of MNPs. Furthermore, the number of incorporated particles is limited because of the interference of the UV light with particles in the hydrogel solution. Therefore, the fabrication process with MNP integration before curing is not used for further experiments. More information about the fabrication process can be found in the Supplementary Methods, as well as MPS and VSM measurements in Supplementary Figs. 1 and 2.

The pH-dependent swelling behavior of magnetic hydrogels with a diameter of approximately 3 mm in the dry state was examined by adding hydrogels to solutions with varying pH values. The difference in size caused by swelling can be observed with the naked eye, as shown in Fig. 2b. Figure 2c illustrates the mean calculated swelling ratio as the percentage increase in weight for six hydrogels. A statistically significant correlation was observed between pH value and swelling ratio, in which the swelling ratio increases with increasing pH levels, thus achieving a maximum swelling ratio of approximately 555% at pH 10. The acidic pH range shows a linear relationship between swelling and pH, leading to a maximum swelling ratio of approximately 146% at pH 7. The discrepancy between the absolute amount of swelling in the acidic pH values and the basic pH value of pH 10 can be explained by the typical swelling behavior of the anionically charged hydrogels. In a basic pH value, strong electrostatic repulsive forces are generated inside the gel, resulting in pronounced swelling in the basic pH range. The linear swelling observed in the acidic range provides evidence that the material is suitable for use in sensing applications within the medical field, as several medical conditions are associated with altered acidic pH values^20^.

### Swelling-induced signal change

**Fig. 3:**
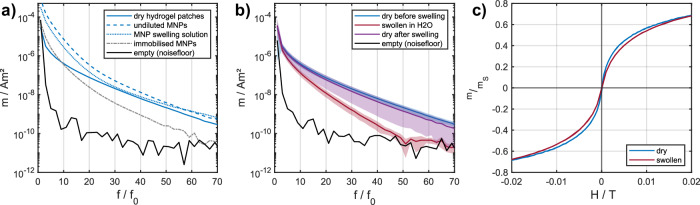
Investigation of the magnetic signal of hydrogel patches in dry and swollen states. **a** Magnetic particle spectrometry (MPS) amplitude spectrum. Characterization of dry magnetic patches in comparison to magnetic nanoparticle (MNP) reference samples (undiluted MNPs, diluted MNP swelling solution, and immobilized MNPs), **b** MPS amplitude spectrum. The same hydrogels are measured in the dry state, in the swollen state, and in the dry state after swelling. Shown is the mean value for 6 patches, and colored corridors indicate the standard deviation. **c** Magnetization curve of a magnetic hydrogel patch in the dry and swollen state. Measured with a vibrating sample magnetometer at a field strength between −20 mT and 20 mT, which corresponds to the excitation amplitude in MPS, normalized to the saturated magnetic moment *m*_*S*_.

Magnetic particle spectrometry (MPS) measurements, performed to investigate the magnetic behavior of the magnetic patches, showed that the MPS amplitude spectrum of dry magnetic hydrogel patches differentiates in the lower harmonics from the MPS spectrum of the same volume of undiluted MNP solution. In higher harmonics, the amplitudes show similarities with the measured MNP reference and the MNP swelling solution used for MNP absorption (compare Fig. 3a). In addition, it can be observed that the signal in the low harmonics intersects the signal from the immobilized MNP reference. This demonstrates that the relaxation behavior of MNPs is influenced by integration in the hydrogel, resulting in a different MPS spectrum. Despite the reduction in the absolute amount of MNPs in the hydrogel compared to that of undiluted MNPs, the magnetic hydrogels demonstrate promising MPI signal characteristics (broad MPS amplitude spectrum up to higher harmonics).

To characterize the magnetic signal behavior of the produced samples in the swollen state, the hydrogel patches are measured in MPS and VSM. The MPS amplitude spectrum in Fig. 3b shows that the amplitudes of the swollen hydrogels exhibit a steeper decline toward the higher harmonics than the amplitudes of the dry hydrogels, reaching a level comparable to that of the empty measurement and the noise level around the 41st harmonic. In comparison to that, dry hydrogels are reaching background noise between the 80th and 90th harmonics. This indicates that the number of detected harmonics is reduced in the swollen state.

The observed signal change can be attributed to a modification of the particle-matrix and particle–particle interactions resulting from the swelling process. The precise interaction between hydrogel swelling and the change in particle relaxation dynamics remains unknown. However, the effect of this change is reproducible and thus can be exploited as a sensing technique for resolving the pH value. One possible underlying cause could be a change in the particle interactions as the swelling of the hydrogel increases the mean distance between the individual particles (interparticle distance). After the swollen hydrogels were measured, the samples were dried again to investigate the reversibility of the process. The results in Fig. 3b show that renewed drying after swelling in water produces a higher MPS signal again, comparable to the signal before swelling, indicating that the MPS amplitude signal can be restored. The reversibility of the particle interactions could provide an explanation for this observation. Further studies will investigate the cause of the dependence of the MNP signal on hydrogel swelling and the resulting signal behavior. It is also important to note that the data obtained from the measurement of the hydrogels in their dry state following the swelling process exhibit a considerable degree of variability in the amplitude spectrum (represented by a wider purple corridor in Fig. 3b). This could be attributed to the loss of MNPs, which should not occur in a real pH monitoring scenario where the hydrogel remains constant in solution and is not exposed to strong differences in swelling. Despite this assumption, this observation raises the question of permanent loss of embedded particles through swelling. Further studies should therefore also focus on the long-term stability of the magnetic hydrogels in different swelling states.

The VSM data shown in Fig. 3c substantiate the aforementioned signal change in the MPS, also for the static magnetization case. The figure illustrates the magnetic moment of one patch as a function of external field strength within the range of an MPS excitation field strength. It is evident that the swelling process results in a modification of the magnetization behavior, which can be discerned in a transformed magnetization curve as observed in the VSM. This alteration can be directly associated with the signal change observed in MPS.

### pH-dependent MPS-signal

**Fig. 4:**
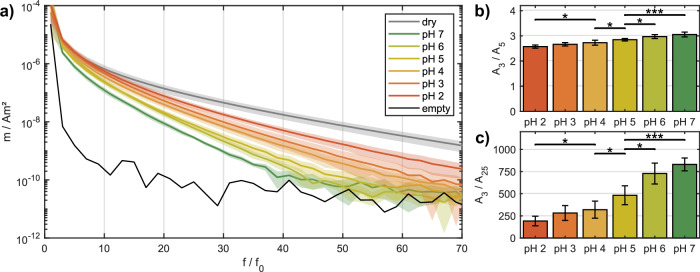
Magnetic particle spectrometry (MPS) measurements of hydrogel patches at different pH values. **a** MPS amplitude spectrum (odd harmonics only). The dry state before swelling and the swollen state for pH values between 2 and 7 are shown. Spectra are calculated as the mean values of 6 samples, and colored corridors indicate the standard deviation. **b** Harmonic ratio of 3rd and 5th and **c** 3rd and 25th harmonic magnitude. The mean value of 6 samples, as well as the standard deviation and statistical significance, are shown.

To investigate the influence of different pH values corresponding to different swelling ratios, the samples are measured in MPS in different pH solutions. Figure 4a illustrates the MPS amplitude spectra of hydrogel patches in solutions with pH values in the acidic range between pH 2 and 7. In addition, the amplitude curves of the dry samples before swelling are presented. The spectra were derived as the mean of six samples. The investigations were carried out within the acidic range, given the significance of the pH range for medical applications^20^.

A decline in the magnetic moment of the spectra from different samples can be observed with an increase in the pH value. The highest signal is generated by hydrogels in the dry state, followed by samples in solution at pH 2. As the pH value increases, a reduction in the spectrum of harmonics becomes evident, with the smallest signal present at a pH of 7. This behavior is consistent with that observed in distilled water (see Fig. 3a), where a swollen state results in a lower signal than a dry state. In addition, this experiment shows that a higher pH value, indicative of a higher swelling ratio, is associated with a reduction in the MPS signal. However, it can also be observed that the values of the individual measurements and the standard deviation corridors partially overlap. This can be attributed to the disparate distribution of particles within different hydrogel patches, which results in varying concentrations. Furthermore, the hydrogels exhibit slight variations in their swelling behavior, which may contribute to the observed differences in spectra.

In order to gain further insight into the diverse signal behavior of patches in different pH solutions, additional metrics describing the MPS signal were defined to examine the characteristics of the lower and higher harmonics in the MPS amplitude spectrum. To this end, the ratio of the third to the fifth harmonic and the ratio of the third to the twenty-fifth harmonic were calculated for each sample. The third harmonic ratio is also used to eliminate the influence of particle concentration in the measured data, as long as there are no particle–particle interactions present. The results of these calculations are presented in Fig. 4b, c, showing that a statistically significant distinction is possible for differences in single pH values between pH 4, 5, and 6. Both the calculated ratio of the low harmonics (Fig. 4b) and that of the higher harmonics (Fig. 4c) demonstrate these differences, in which the harmonic ratio increases with each pH value. This trend in the MPS signal enables a statistically significant distinction to be made between larger pH ranges of 2 and 4 and between 5 and 7. As a result, these pH values, pH 2, 4, and 7, were selected for multi-contrast MPI measurements. Future investigations will also focus on the dynamic signal behavior by measuring the same hydrogel patch in different pH solutions to simulate a pH change. For the diagnosis of pH-related diseases, the pH resolution should also be further investigated in a pH range around pH 7 of ±0.5, which represents a critical threshold value for early detection. In addition, MNPs that are not embedded in the hydrogel should be measured in solutions with different pH values to investigate the influence of the pH value on the particles themselves.

### pH-resolving in multi-contrast MPI

**Fig. 5:**
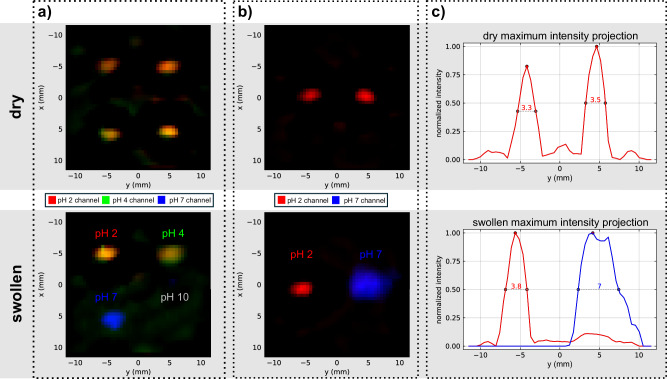
Reconstructed images of performed magnetic particle imaging measurements of magnetic hydrogels in different pH solutions. First row: samples in dry state, second row**:** same samples in swollen state. Images in (**a**) are reconstructed with three system matrices with multi-color mapping with red pH 2 channel, green pH 4 and blue pH 7 channel. Images in (**b**) are reconstructed with two system matrices for pH 2 and 7. (**c**) shows maximum intensity projections of (**b**) with calculated full-width at half-maximum values.

To demonstrate the feasibility of resolving different pH values with pH-responsive magnetic hydrogels in an MPI image, measurements were performed in a commercial preclinical MPI scanner (Bruker MPI System 25/20 FF). Figure 5 shows the reconstructed images of measurements performed with magnetic hydrogels in different pH solutions. The images in the first row contain the samples in the dry state before immersion in the pH solution. Figures in the second row show the same samples in pH solutions with pH 2, 4, 7, and 10 in the swollen state. In the images in Fig. 5a, reconstruction is performed using a multi-contrast approach with three system matrices for pH 2, 4, and 7, merging the results into one image using different colors for each pH channel. Image reconstruction with two system matrices for pH 2 and 7 is shown in the images in Fig. 5b with hydrogels in pH 2 and 7. In Fig. 5c, the maximum intensity is shown along the y-axis of the images in Fig. 5b, where two peaks mark the position of the hydrogels. In addition, the corresponding full-width at half-maximum (FWHM) values are calculated for every peak. After measuring the system matrices for pH 2 and 4, a misalignment of the system matrix patterns was observed, which is probably due to a movement of the samples during the acquisition of the system matrix. This misalignment is corrected by moving the center of the system matrices until they align. The uncorrected images can be found in Supplementary Fig. 3.

All images in the swollen states show differences in the signal intensity of different pH channels compared to the images in the dry states of the hydrogels. Reconstruction with three system matrices in Fig. 5a enables differentiation of pH values 2, 4, and 7. The samples in pH 2 and 4 show the signal of both pH 2 and 4 channels. However, the hydrogel in pH 2 has a larger contribution of the pH 2 channel, and pH 4 has a larger contribution of the pH 4 channel (compare separate subplots for each pH channel in Supplementary Fig. 4). Furthermore, the hydrogel at pH 10 does not generate any visible signal in the image reconstruction. One possible explanation for the missing signal could be that the signal strength between pH 2 and 10 differs too much to be calculated with the present approach, see ref. ^32^. In addition, the missing system matrix for a pH 10 channel could lead to the missing signal in the image.

When the sizes of the hydrogels in the image are compared, there seems to be no difference, which does not fit the real size proportions of the gels. The sizes of hydrogels in the dry and swollen state were measured as the swelling behavior was investigated. The diameters were averaged for the dry state with ~3.1 mm, for pH 2 with ~3.5 mm, for pH 4 with ~4.2 mm, for pH 7 with ~6.6 mm, and for pH 10 with ~7.8 mm. However, we found that the evaluation of the swelling in terms of weight is more precise, which is why we did not perform any further size assessment of the swelling process. When comparing the reconstruction with only two system matrices in Fig. 5b, a clear difference in size can be observed between pH 2 and 7. Although all images were calculated with the same parameters, the reconstruction results in these different sizes for the pH 7 hydrogel. However, the systems of equations to solve the reconstruction problem differ in their scope and complexity when adding another system matrix, which could have led to different sizes for the hydrogel at pH 7 when using two or three system matrices for reconstruction. In general, note that there may be a distortedIn the PDF Version, Figure 1 should be inserted directly in the corresponding subsection "Principle of resolving pH" like in the online version, as it gives an introduction to the working principle. representation of the sizes of the hydrogels because the system matrices were not recorded with point samples but with whole hydrogels of different sizes. Therefore, errors in the reconstructed sizes are to be expected.

In reconstructed images with only two system matrices in pH 2 and 7 (Fig. 5b), the swelling process is visible in different sizes and in the FWHM values of the maximum intensity projections (Fig. 5c). The hydrogel at pH 7 shows an FWHM almost double the size of the hydrogel at pH 2 and aligns with the measured sizes of the swollen hydrogels. Future studies will examine in more detail whether the method is suitable for determining the actual size of hydrogels. In addition, a small leakage of the pH 2 channel can be observed in the pH 7 channel; compared to the pH 2 channel, pH 7 does not show any channel leakage. In the end, the successful reconstruction of different pH values is demonstrated by showing possible pH differentiation between pH 2, 4, and 7. Like for MPS, further investigation will address the dynamic behavior when the pH of the solutions is changed and the resolution of differentiating pH values.

## Conclusions

In this proof-of-concept work, we resolved the pH in multi-contrast MPI by indirectly generating pH contrast using pH-dependent swelling of a hydrogel to modulate the signal of embedded MNPs. The successful production of pH-responsive magnetic hydrogel patches was demonstrated by photopolymerization of the copolymer hydrogel PHEMA-AA and integration of MNPs through immersion of the cured patches in a solution containing Synomag-D MNP. These patches exhibit a strong magnetic signal in the dry state and linear pH-dependent swelling behavior in the acidic range. The MPS amplitude spectra show a negative correlation with the pH-dependent swelling ratio of the magnetic hydrogel patches. The linear relationship between the swelling ratio and the pH in the acidic pH range facilitates the assignment of a specific MPS signal to a specific pH value. The trend observed across all investigated pH values indicates that higher pH values, indicative of higher swelling ratios, are associated with a lower MPS signal. The results of MPI measurements demonstrate the feasibility of resolving pH in the MPI image using a multi-contrast reconstruction approach. Reconstruction in three different channels with three different system matrices enables differentiation between pH values 2, 4, and 7.

Further experiments will address the dynamic investigation of patches in which a change in the pH value is time-dependently identified in a changed MPI signal. Furthermore, to determine the precision of pH value resolving, the pH value distance between the tested solutions should be minimized. In particular, the narrow range around pH 7 of ±0.5 represents a critical threshold value for the early detection of pH-related diseases. If these pH deviations manifest as discernible variations in the measurement signal, the employment of pH-dependent magnetic hydrogel patches would enable precise pH detection for comprehensive diagnostic assessments using MPI. This enhancement would extend the feasible range of measurement variables in multi-contrast MPI to include pH values, thereby enabling non-invasive pH monitoring for a wide range of biomedical applications. Among these applications are the investigation of inflammation-related pH alterations and the assessment of the tumor microenvironment through pH value measurements. The latter encompasses the development of smaller magnetic gels, as hydrogels at a microscopic scale may be utilized to permeate the tumor microenvironment within in vivo studies.

## Methods

### Magnetic hydrogel patch fabrication

The polyacrylate hydrogel used in this work is the synthetic copolymer hydrogel 5-poly(2-hydroxyethyl methacrylate-co-acrylic acid) (PHEMA-AA) made of hydroxyethyl methacrylate (HEMA) and acrylic acid (AA). For all experiments, cylindrical hydrogel patches have been manufactured with a cylindrical mold with a diameter of 2.5 mm and a depth of 1 mm using photopolymerization.

The composition of the hydrogel comprises the monomers HEMA and AA, in addition to the photoinitiator 2-dimethoxy-2-phenylacetophenone (DMPA) and the crosslinker ethylene glycol dimethacrylate (EDGMA). All chemicals were obtained from Merck (Merck KGaA, Darmstadt, Germany) and used as received. At first, 819 μl of HEMA with 30 mg of DMPA are combined in a 1.5 ml Eppendorf tube and briefly mixed using a vortex. Subsequently, 18 μl of EDGMA and 462 μl of acrylic acid are added, and the solution is mixed using the vortex for a period of 10 min and ultrasonic agitation at 40 kHz for 20 min, thus ensuring homogeneous mixing of the components. A silicone mold to form the patches was manufactured through a mold-casting process using a 3D-printed master mold. The hydrogel solution is pipetted into this silicone mold (7.5 μl per patch) and photopolymerization takes place by irradiating the patches with a 100 watt UV light LED panel at a distance of 5 cm. Following a period of irradiation between 3 and 4 min, the hydrogels are removed and washed initially in ethanol and subsequently in distilled water. This procedure is followed by storage of the hydrogels in distilled water for several days to facilitate the removal of any residual components that are not bound during curing. Finally, the patches are dried at room temperature for another 24 h.

**Fig. 6:**
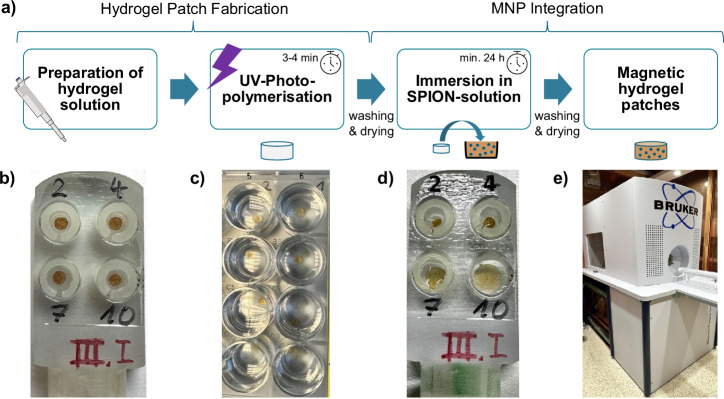
Magnetic hydrogel patch fabrication and preparation of hydrogels for MPI in a 3D-printed phantom. **a** Fabrication process: After preparation of the hydrogel solution, hydrogel patches are UV-cured by photopolymerization. Then the patches are immersed in superparamagnetic iron oxide nanoparticles (SPION) solution for integrating magnetic nanoparticles through absorption into the hydrogel matrix. **b** Phantom filled with dry magnetic hydrogel patches. **c** Swelling of the hydrogels for 24 h in a 24-well plate. **d** Phantom filled with swollen hydrogels in pH 2, 4, 7, and 10. **e** Used preclinical MPI System (Bruker MPI system 25/20 FF).

Integration of MNPs into the hydrogel takes place by immersion of the dry hydrogel patches in a SPION solution. The SPION solution consists of Synomag-D particles (50 nm, micromod Partikeltechnologie GmbH, Rostock, Germany) mixed with destilled water in a dilution ratio of 1:3, which corresponds to a particle concentration of 8.3 mg/ml. The hydrogels are kept in the particle solution for at least 24 h, after which they are removed, washed in distilled water, and dried at room temperature for 24 h. An overview of the fabrication process can be found in Fig. 6a. An alternative method for MNP integration involves the mixing of MNPs with the liquid hydrogel solution prior to UV curing. This approach is further described in the Supplementary Methods section.

### Investigation of pH-dependent swelling behavior

In order to investigate the swelling behavior and perform MPS and MPI measurements with hydrogels at different pH values, solutions of sodium hydroxide and hydrochloric acid at the appropriate pH levels (pH 2, 3,…, 7 and 10) are prepared by diluting monomolar stock solutions. The pH value is checked with a pH meter (Mettler Toledo International Inc., Columbus, USA). To quantify the pH-dependent swelling of the hydrogels, the hydrogels are first weighed in the dry state (*W*_0_). After keeping them in solutions for 24 h, the swelling ratio is then calculated by weighing the gels in the swollen state (*W*_*S*_), with:

1 $$Swelling\,ratio\,( \% )\,=\,\frac{{W}_{s}-{W}_{0}}{{W}_{0}}\cdot 100.$$

In that way, the mean swelling ratio is determined for multiple samples, representing the percentage increase in weight for different pH values. Statistical significance is calculated using the one-sample *t*-test and is shown with asterisks, where * = *p *≤ 0.05, ** = *p *≤ 0.01, and *** = *p *≤ 0.001. A more detailed investigation of swelling behavior is conducted within the acidic pH range between pH 2 and 7, with a pH value difference of one, due to the relevance of the acidic range for the diagnosis of pH-altering diseases^20^.

### MPS and VSM measurements

For characterization of the magnetic hydrogel patches, distinct samples are first measured in the dry state in MPS and compared to the MNP reference samples. Reference samples consist of undiluted Synomag-D, the swelling solution of MNPs used to manufacture the patches, and immobilized Synomag-D (immobilized with the NanoSeal 180 W) impregnating agent (JELN Imprägnierung GmbH, Schwalmtal, Germany). To investigate the swelling-related signal behavior of distinct samples, they are first measured with MPS and vibrating sample magnetometry (VSM) in the dry and swollen state by immersing them in distilled water for 24 h. Subsequently, the hydrogel patches were subjected to a second drying process to ascertain whether the signal dependence on the swelling ratio is reversible and whether the initial signal can be restored through renewed drying. MPS measurements were always performed after the hydrogel sample was removed from the liquid. Therefore, any freely moving MNPs from the surrounding solution do not contribute to the measured particle signal. To prevent spatial shift of the samples in the measurement field due to different sample volumes, the tip of the Eppendorf tubes used was filled with 50 μl epoxy resin and cured. This procedure ensures that all samples are measured in the same position in the measuring coil. Second, the MPS signal is determined for hydrogel patches in solutions with different pH values, resulting in different swelling ratios of the gels. Therefore, patches are immersed in solutions with pH values between 2 and 7 for 24 h and then measured in different swollen states in MPS.

The one-dimensional MPS used in this work was developed by Biederer et al.^4^. Measurements are carried out with a magnetic field strength of 20 at an excitation frequency of 25 kHz. Ten frames are recorded for each measurement. Each frame in turn corresponds to an averaging over 0.4 s.

Measurements in the VSM are performed with the 8600 Series VSM from Lake Shore Cryotronics Inc. (Ohio, USA). The applied magnetic field strength is continuously swept between 2 and −2. A sampling rate of 1 is used in the range between −100 and 100, and outside the range, a sampling rate of 40 is used to record the resulting magnetic moment of the sample. In order to better compare the magnetic moments of samples with different signal strengths, all measurements are normalized to their saturated magnetic moment.

### MPI measurements and multi-contrast reconstruction

For all MPI measurements, the preclinical Bruker MPI system 25/20 FF (Bruker BioSpin GmbH & Co. KG, Ettlingen, Germany) is used (Fig. 6e). The excitation of the samples is performed with a 2D Lissajous trajectory in the xy direction with an amplitude of 12 at a frequency of 24.509 kHz and 26.041 kHz in the x and y directions, respectively, and a selection field gradient of 1.2 Tm^−1^, generating a FOV of 20 mm × 20 mm. The measurements are averaged over 1 s.

For improved sensitivity, a dedicated receive coil based on the gradiometric approach is used, as described by Graeser et al.^33^. For multi-contrast reconstruction, three 2D system matrices are acquired with magnetic hydrogel patches in solutions with pH 2, 4, and 7 with a grid of 32 × 32 pixels and a width of 0.75 mm per voxel. Because of the unavailability of precise iron concentration values in the hydrogels, samples with equivalent MPS amplitude spectra were selected to ensure comparable particle concentration within each hydrogel. Image reconstruction is performed using an iterative Kaczmarz algorithm with the same reconstruction parameters for all images^34^. Reconstruction parameters are selected to optimize the generated image. In addition, the values for an image with a grid of 48 × 48 pixels are interpolated from the 32 × 32 pixel measurements. For reconstruction, a signal-to-noise threshold of 10, a minimum frequency of 27 kHz, 100 iterations, and an L2-regularization strength of 0.05 are used as reconstruction parameters. In addition, background correction is performed with dedicated empty measurements for every image. First, dry hydrogels are placed in a 3D-printed phantom with 4 holes (Fig. 6b) and measured. Inserts with different inner diameters are used to ensure centering of the gels. After immersing the hydrogels for 24 h in solutions with pH values of 2, 4, 7, and 10 using a 24-well plate and 2.5 ml solution per well (Fig. 6c), the samples are transferred back to the phantom and measured in the swollen state. The holes are then filled with different pH solutions to prevent drying and loss of hydrogel volume during measurement (Fig. 6d) and then measured in the MPI system (Fig. 6e).

## Supplementary information

Supplementary Information

## Data Availability

All the data needed to evaluate the findings in this article are present in the article and/or in the Supplementary Information. Additional data related to this paper can be requested from the corresponding authors via email.

## Code availability

The code used to perform the MPS measurements is publicly available on GitHub. You can access the code repository via the following link: https://github.com/MagneticParticleImaging/MPIMeasurements.jl. The code used for multi-contrast image reconstruction can also be accessed via the following link: https://github.com/MagneticParticleImaging/MPIReco.jl. The acquired MPI dataset and detailed reconstruction parameters are available upon reasonable request.

## References

[1] Gleich, B. & Weizenecker, J. Tomographic imaging using the nonlinear response of magnetic particles. *Nature* **435**, 1214–1217 (2005). 15988521 10.1038/nature03808

[2] Knopp, T., Gdaniec, N. & Möddel, M. Magnetic particle imaging: from proof of principle to preclinical applications. *Phys. Med. Biol.* **62**, R124 (2017). 28398219 10.1088/1361-6560/aa6c99

[3] Weaver, J. B., Rauwerdink, A. M., Sullivan, C. R. & Baker, I. Frequency distribution of the nanoparticle magnetization in the presence of a static as well as a harmonic magnetic field. *Med. Phys.* **35**, 1988–1994 (2008). 18561675 10.1118/1.2903449 PMC4108637

[4] Biederer, S. et al. Magnetization response spectroscopy of superparamagnetic nanoparticles for magnetic particle imaging. *J. Phys. D Appl. Phys.* **42**, 205007 (2009).

[5] Rauwerdink, A. M., Hansen, E. W. & Weaver, J. B. Nanoparticle temperature estimation in combined ac and dc magnetic fields. *Phys. Med. Biol.* **54**, L51 (2009). 19741275 10.1088/0031-9155/54/19/L01 PMC3757125

[6] Rahmer, J., Halkola, A., Gleich, B., Schmale, I. & Borgert, J. First experimental evidence of the feasibility of multi-color magnetic particle imaging. *Phys. Med. Biol.* **60**, 1775 (2015). 25658130 10.1088/0031-9155/60/5/1775

[7] Stehning, C., Gleich, B. & Rahmer, J. Simultaneous magnetic particle imaging (MPI) and temperature mapping using multi-color MPI. *Int. J. Magn. Part. Imaging* **2**, 2 (2016).

[8] Möddel, M., Meins, C., Dieckhoff, J. & Knopp, T. Viscosity quantification using multi-contrast magnetic particle imaging. *N. J. Phys.* **20**, 083001 (2018).

[9] Utkur, M., Muslu, Y. & Saritas, E. U. Relaxation-based color magnetic particle imaging for viscosity mapping. *Appl. Phys. Lett.* **115**, 152403 (2019).

[10] Shasha, C. et al. Discriminating nanoparticle core size using multi-contrast MPI. *Phys. Med. Biol.* **64**, 074001 (2019). 30870817 10.1088/1361-6560/ab0fc9

[11] Möddel, M., Griese, F., Kluth, T. & Knopp, T. Estimating the spatial orientation of immobilized magnetic nanoparticles with parallel-aligned easy axes. *Phys. Rev. Appl.* **16**, L041003 (2021).

[12] Paysen, H. et al. Cellular uptake of magnetic nanoparticles imaged and quantified by magnetic particle imaging. *Sci. Rep.* **10**, 1922 (2020). 32024926 10.1038/s41598-020-58853-3 PMC7002802

[13] Draack, S. et al. Determination of dominating relaxation mechanisms from temperature-dependent magnetic particle spectroscopy measurements. *J. Magn. Magn. Mater.* **474**, 570–573 (2019).

[14] Draack, S., Schilling, M. & Viereck, T. Magnetic particle imaging of particle dynamics in complex matrix systems. *Phys. Sci. Rev.* **8**, 213–237 (2023).

[15] Szwargulski, P. et al. Monitoring intracranial cerebral hemorrhage using multicontrast real-time magnetic particle imaging. *ACS Nano* **14**, 13913–13923 (2020). 32941000 10.1021/acsnano.0c06326

[16] Thieben, F. et al. System characterization of a human-sized 3D real-time magnetic particle imaging scanner for cerebral applications. *Commun. Eng.* **3**, 47 (2024).

[17] Haegele, J. et al. Multi-color magnetic particle imaging for cardiovascular interventions. *Phys. Med. Biol.* **61**, N415 (2016). 27476675 10.1088/0031-9155/61/16/N415

[18] Rahmer, J., Wirtz, D., Bontus, C., Borgert, J. & Gleich, B. Interactive magnetic catheter steering with 3-D real-time feedback using multi-color magnetic particle imaging. *IEEE Trans. Med. Imaging* **36**, 1449–1456 (2017). 28287965 10.1109/TMI.2017.2679099

[19] Ahlborg, M. et al. First dedicated balloon catheter for magnetic particle imaging. *IEEE Trans. Med. Imaging* **41**, 3301–3308 (2022). 35709119 10.1109/TMI.2022.3183948

[20] Hajjar, S. & Zhou, X. pH sensing at the intersection of tissue homeostasis and inflammation. *Trends Immunol.* **44**, 807–825 (2023). 10.1016/j.it.2023.08.008 PMC10543622 37714775

[21] Kuo, S.-H., Shen, C.-J., Shen, C.-F. & Cheng, C.-M. Role of pH value in clinically relevant diagnosis. *Diagnostics* **10**, 107 (2020). 32079129 10.3390/diagnostics10020107 PMC7167948

[22] Wichterle, O. & Lim, D. Hydrophilic gels for biological use. *Nature* **185**, 117–118 (1960).

[23] Mahinroosta, M., Farsangi, Z. J., Allahverdi, A. & Shakoori, Z. Hydrogels as intelligent materials: a brief review of synthesis, properties and applications. *Mater. Today Chem.* **8**, 42–55 (2018).

[24] Deligkaris, K., Tadele, T. S., Olthuis, W. & van den Berg, A. Hydrogel-based devices for biomedical applications. *Sens. Actuators B Chem.* **147**, 765–774 (2010).

[25] Ullah, F., Othman, M. B. H., Javed, F., Ahmad, Z. & Akil, H. M. Classification, processing and application of hydrogels: a review. *Mater. Sci. Eng. C* **57**, 414–433 (2015). 10.1016/j.msec.2015.07.053 26354282

[26] Feng, W. & Wang, Z. Tailoring the swelling-shrinkable behavior of hydrogels for biomedical applications. *Adv. Sci.* **10**, 2303326 (2023). 10.1002/advs.202303326 PMC10558674 37544909

[27] Gil, E. S. & Hudson, S. M. Stimuli-reponsive polymers and their bioconjugates. *Prog. Polym. Sci.* **29**, 1173–1222 (2004).

[28] Pinelli, F., Magagnin, L. & Rossi, F. Progress in hydrogels for sensing applications: a review. *Mater. Today Chem.* **17**, 100317 (2020).

[29] Lavrador, P., Esteves, M. R., Gaspar, V. M. & Mano, J. F. Stimuli-responsive nanocomposite hydrogels for biomedical applications. *Adv. Funct. Mater.* **31**, 2005941 (2021).

[30] Arifuzzaman, M. et al. An implanted pH sensor read using radiography. *Analyst* **144**, 2984–2993 (2019). 30888348 10.1039/c8an02337a PMC6491216

[31] Richter, A. et al. Review on hydrogel-based pH sensors and microsensors. *Sensors* **8**, 561–581 (2008). 27879722 10.3390/s8010561 PMC3668326

[32] Boberg, M. et al. Simultaneous imaging of widely differing particle concentrations in MPI: problem statement and algorithmic proposal for improvement. *Phys. Med. Biol.* **66**, 095004 (2021). 10.1088/1361-6560/abf202 33765669

[33] Graeser, M. et al. Towards picogram detection of superparamagnetic iron-oxide particles using a gradiometric receive coil. *Sci. Rep.* **7**, 6872 (2017). 28761103 10.1038/s41598-017-06992-5 PMC5537232

[34] Knopp, T. et al. Mpireco.jl: Julia package for image reconstruction in MPI. *Int. J. Magn. Part. Imaging* **5**, 9 (2019).

