# Supplementary material for: Multi-contrast magnetic particle imaging for tomographic pH monitoring using stimuli-responsive hydrogels
Source: Commun Eng. 2026 Jan 17;5:33. doi: 10.1038/s44172-026-00586-8 (PMC12909995; doi:10.1038/s44172-026-00586-8)
Supplement: Supplementary file 1 — Supplementary Information [file 44172_2026_586_MOESM1_ESM.pdf]

Multi-contrast magnetic particle imaging for tomographic pH monitoring using stimuli-responsive hydrogels: Supplementary Information

Bruno Kluwe[1,2*], Justin Ackers[1], Matthias Graeser[1,3], Anna C. Bakenecker[1,4*]

[1]Fraunhofer IMTE, Fraunhofer Research Institution for Individualized and Cell-Based Medical Engineering, Lübeck, Germany.
[2]Physikalisch-Technische Bundesanstalt, Metrology for Magnetic Nanoparticles, Berlin, Germany.
[3]Chair for Metrology, University of Rostock, Rostock,Germany.
[4]Medical Engineering, Department of Electrical Engineering and Information Technology, Technical University of Darmstadt, Darmstadt, Germany.

*Corresponding author(s). E-mail(s): bruno.kluwe@ptb.de; anna.bakenecker@tu-darmstadt.de;
Contributing authors: justin.ackers@imte.fraunhofer.de; matthias.graeser@imte.fraunhofer.de;

Abstract

Magnetic particle imaging (MPI) is a tomographic imaging technique which determines the spatial distribution of magnetic nanoparticles (MNPs). Multi-contrast MPI provides the ability to detect environmental conditions of MNPs, such as temperature or viscosity. One parameter that has not been investigated but shows high potential for medical diagnosis is the pH value, as it is an indicator of inflamed or tumorous tissue. In this work, we present an approach to resolve the pH value using multi-contrast MPI. Our proof-of-concept is based on a stimuli-responsive, magnetic hydrogel that exhibits reversible swelling in response to a pH change. The pH contrast is generated indirectly via the pH-responsive hydrogel swelling modulating the signal of embedded MNPs. Magnetic particle spectrometry measurements show that the hydrogels' magnetic response

correlates with the pH value, which could provide a new way of contactless pH monitoring. Finally, the feasibility of resolving different pH values in a multi-contrast MPI image is demonstrated.

Supplementary Information

Supplementary methods: Alternative magnetic hydrogel patch fabrication

In addition to the method described in the methods part ”magnetic hydrogel patch fabrication”, an alternative approach to fabricating magnetic hydrogel patches involves the incorporation of MNPs into the hydrogel solution prior to UV cure. The optimal particle concentration is determined by introducing varying concentrations into the hydrogel solution. The findings demonstrate that low concentrations yield insufficient signal strength, while high concentrations influence the curing process, resulting in uneven cured gels with low mechanical stability. Synomag-D, at a concentration of 1.7 mg/ml in a PHEMA-AA solution, has been demonstrated to produce uniform hydrogels with a measurable magnetic signal. The MNPs are added to the hydrogel solution and subsequently mixed using an ultrasonic homogenizer for a duration of 20 minutes at a power output of 20 %. Compared to a hydrogel solution without MNPs, the curing time is increased to approximately eight minutes.

Supplementary Note 1: Signal comparison of different MNP-Integration

Supplementary Figure 1 shows the results of MPS measurements of a magnetic hydrogel patch with MNP-integration before curing compared to a patch with MNP-integration after curing. In addition, reference samples consisting of undiluted liquid Synomag (Synomag reference with 25 mg/ml Synomag-D) and immobilized Synomag with the same volume as the hydrogel (4.5 µl) were measured.

The spectrum of the patch with MNP-integration before curing shows a weaker signal than the patch with MNP-integration after the curing process. In direct comparison, the amplitudes are approximately one order of magnitude lower, reaching the noise level at the 40th harmonic compared to the 80th harmonic for MNP integration after curing. Further, it can be observed that the MPS amplitude spectrum of hydrogels with MNP-integration after curing differentiates in the lower harmonics from the MPS spectrum of the MNP reference sample. In higher harmonics, the amplitudes show similarities with the undiluted MNP. The spectrum of the patch with MNP-integration before curing is similar to the spectrum of immobilized Synomag (small shift due to a lower amount of MNPs), immobilised with NanoSeal 180 W impregnating agent (JELN Imprägnierung GmbH, Schwalmtal, Germany). This observation is consistent with the hypothesis that the curing of hydrogels with MNPs inside immobilizes the MNPs, thereby leading to a reduced magnetic signal and therefore lower amplitudes in the MPS spectrum.

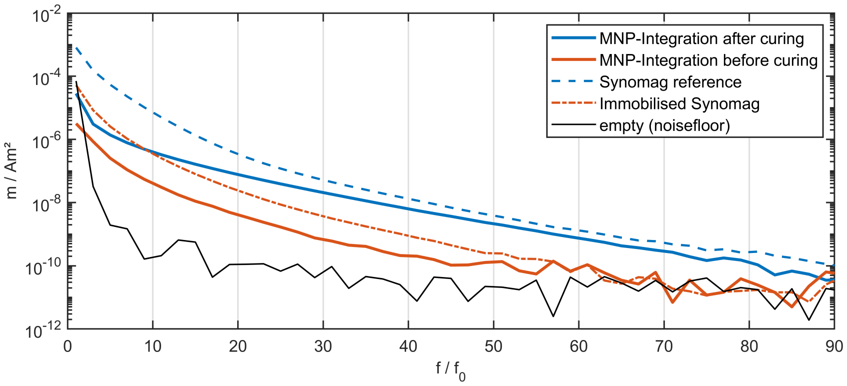

Supplementary Figure 1: MPS amplitude spectrum of the differently produced hydrogel patches with reference samples. The figure shows measurements from one sample for each spectrum. Synomag reference consists of 4.5 µl solution with 25 mg/ml Synomag-D. Immobilized Synomag sample consists of 4.5 µl Synomag-D cured with Nanoseal impregnating agent.

Supplementary Note 2: Additional MPS- / VSM-Measurements in swollen state

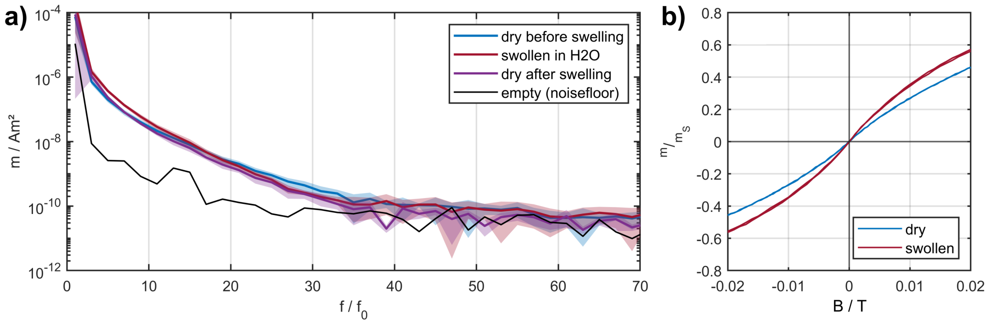

Supplementary Figure 2: Investigation of the magnetic signal of hydrogel patches with MNP-Integration before curing in the dry and swollen state. **a):** MPS amplitude spectrum. Same hydrogels are measured in the dry state, in swollen state in destilled water and in the dry state after swelling. Shown is the mean value for 6 patches and colored corridors indicate the standard deviation. **b):** Magnetization curve of a magnetic hydrogel patch in the dry and swollen state. Measured in VSM, shown in the MPS excitation range between −20 mT and +20mT, normalized to saturated magnetic moment m_S.

Supplementary Figure 2a shows the MPS amplitude spectrum of magnetic hydrogel patches with integration of MNP before curing in the dry and swollen state.

In contrast to the manufacturing process with particle integration after curing, the fabricated samples (as described in supplementary methods) show a different signal behavior. It can be observed that the amplitudes of the swollen state are slightly higher than the dry values. This is the case between the third and thirteenth harmonics. For higher harmonics, the amplitude values overlap. In the VSM (Supplementary Figure 2(b)), differences can be seen between the dry and swollen states. Accordingly, the swollen state shows a steeper, nonlinear course of the magnetic moment compared to the dry state. This shows that particle integration strongly influences the signal behavior. In the case of particle integration before curing, the particles are probably more firmly bound to the hydrogel matrix. The behavior in MPS and VSM can be attributed to the swelling and absorption of water into the hydrogel matrix. This is because the Brown relaxation of SPIONs depends on the surface friction of the particles and the surrounding matrix. The swelling of the hydrogel probably leads to reduced friction, enabling the particles to move more freely and generate more signal. Re-drying increases the surface friction of the particles, and the generated magnetic signal decreases again.

Supplementary Figure: MPI images without correcting for system matrix shift

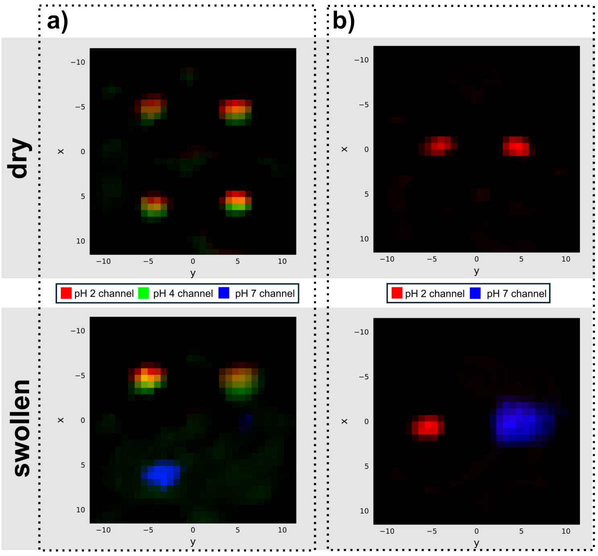

Supplementary Figure 3: Reconstructed MPI images of magnetic hydrogels without system matrix shift correction **First row:** samples in dry state and **second row:** samples in swollen state. Images in **a)** were reconstructed with 3 system matrices with multi color mapping with red pH 2 channel, green pH 4 and blue pH 7 channel. Images in **b)** reconstructed with 2 system matrices for pH 2 and 7.

Supplementary Figure: Multi-contrast subplots for separate pH channels

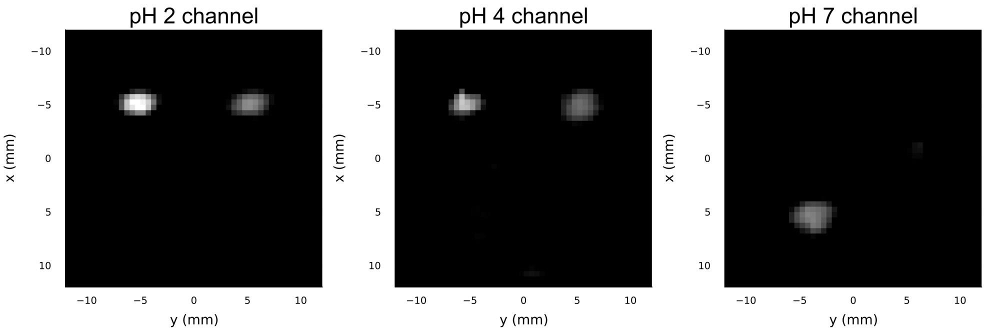

Supplementary Figure 4: MPI images of magnetic hydrogels in pH values 2, 4, 7 and 10 reconstructed with different system matrices. From left to right: reconstructed with system matrix pH 2 (pH 2 channel), reconstructed with system matrix pH 4 (pH 4 channel) and reconstructed with system matrix pH 7 (pH 7 channel).
